# Deep UV Formation of Long-Term Stable Optical Bragg Gratings in Epoxy Waveguides and Their Biomedical Sensing Potentials

**DOI:** 10.3390/s21113868

**Published:** 2021-06-03

**Authors:** Steffen Hessler, Marieke Rüth, Horst-Dieter Lemke, Bernhard Schmauss, Ralf Hellmann

**Affiliations:** 1 Applied Laser and Photonics Group, Faculty of Engineering, University of Applied Sciences Aschaffenburg, Würzburger Straße 45, 63743 Aschaffenburg, Germany; ralf.hellmann@th-ab.de; 2 eXcorLab GmbH, Industrie Center Obernburg, 63784 Obernburg am Main, Germany; marieke.rueth@excorlab.de (M.R.); horstdieter.lemke@excorlab.de (H.-D.L.); 3 Institute of Microwaves and Photonics, University of Erlangen-Nuremberg, Cauerstraße 9, 91054 Erlangen, Germany; bernhard.schmauss@fau.de

**Keywords:** optical sensor, Bragg grating, EpoCore, excimer laser, refractive index sensing, blood analysis, human serum albumin, rapid test

## Abstract

In this article, we summarize our investigations on optimized 248 nm deep ultraviolet (UV) fabrication of highly stable epoxy polymer Bragg grating sensors and their application for biomedical purposes. Employing m-line spectroscopy, deep UV photosensitivity of cross-linked EpoCore thin films in terms of responding refractive index change is determined to a maximum of Δn = + (1.8 ± 0.2) × 10^−3^. All-polymer waveguide Bragg gratings are fabricated by direct laser irradiation of lithographic EpoCore strip waveguides on compatible Topas 6017 substrates through standard +1/-1-order phase masks. According near-field simulations of realistic non-ideal phase masks provide insight into UV dose-dependent characteristics of the Bragg grating formation. By means of online monitoring, arising Bragg reflections during grating inscription via beforehand fiber-coupled waveguide samples, an optimum laser parameter set for well-detectable sensor reflection peaks in respect of peak strength, full width at half maximum and grating attenuation are derived. Promising blood analysis applications of optimized epoxy-based Bragg grating sensors are demonstrated in terms of bulk refractive index sensing of whole blood and selective surface refractive index sensing of human serum albumin.

## 1. Introduction

Over the past two decades, Bragg grating technology has emerged as an attractive optical sensor tool enabling versatile multi-physical parameter monitoring [1,2,3,4]. On the one hand, optical glass fiber Bragg grating (FBG) based sensors are frequently applied in the fields of mechanical structural health monitoring and temperature sensing, confirming commercial application maturity [5,6,7,8]. In addition, Bragg grating sensors created in polymer optical fibers (POFs) have widely been demonstrated, e.g., for popular biomedical healthcare applications [9,10]. On the other hand, a multitude of highly sensitive planar Bragg grating (PBG) based sensing concepts, typically in the form of silica and silicon integrated optical waveguide chips, have been investigated for most diverse application scenarios. By utilizing silicon nano-waveguide Bragg grating structures, on-chip and small footprint temperature sensors have been developed [11]. Directly laser-written PBGs realized in doped fused silica layers were efficiently employed as refractive index sensors for fermentation monitoring, concentration measurements of binary mixtures, and for biomedical substance detection using antibody surface layers [12,13,14,15]. Additionally, significant chemosensory potential for trace gas detection of harmful organic compounds [16] and ozone depleting trichlorofluoromethane was demonstrated using silica-based PBG sensors which were surface functionalized with cyclodextrin capture coatings [17,18].

Since a material substitution by optical polymers, instead, is in general accompanied by notable advantages such as biocompatibility as well as less intricate and costly fabrication procedures besides broadly tunable optical, thermal, and mechanical properties [19,20], polymer-based PBG designs suggest further appealing and particularly inexpensive sensor solutions beyond the material limits of silica and silicon. For instance, two-dimensional mechanical strain sensing as well as humidity insensitive shape sensing were achieved with polymer Bragg gratings in bulk polymethylmethacrylate (PMMA) and cyclic olefin copolymer (COC) substrates [21,22]. Moreover, the cross-linked optical epoxy polymer negative photoresist EpoCore (micro resist technology) turned out to offer exceptional material properties such as high mechanical and extreme chemical robustness [23] compared with COC and PMMA. Owing to EpoCore’s ultraviolet (UV) photosensitivity, precise waveguide geometries can be defined by standard 365 nm photolithography processes. The cured epoxy resist provides polymer-typical optical transparency windows around the primary wavelengths 850 nm, 1310 nm, and 1550 nm [24]. Numerous optomechanical sensing concepts such as freestanding sensor foils with embedded EpoCore waveguide grating structures [25] or flexible carrier substrates with one-side EpoCore strip waveguides for 1550 nm [26] and 850 nm operation [27] have been demonstrated. EpoCore Bragg gratings, furthermore, maintain attractive potential for integrated optical sensing in low-cost biochemical lab-on-a-chip applications [28], not least because of their proven biocompatibility to human blood [29]. In this context, a substantially enhanced refractive index sensitivity of EpoCore Bragg gratings was recently attained by high-refractive TiO_2_ coatings empowering biomedical sensing applications [30].

In this work, we comprehensively investigate the formation and optimization of EpoCore polymer waveguide Bragg gratings by direct excimer laser deep UV irradiation through +1/−1 phase masks and demonstrate promising basic applications for biomedical rapid-testing purposes. Generally, a simple Bragg grating element is formed by a uniform periodic modulation of a waveguide’s refractive index (RI), leading to a spectral narrowband reflection signal at the constructively interfering Bragg wavelength $\lambda_{B}$ according to:

(1) $$m\;\lambda_{B}=2\;n_{\mathrm{eff}}\;\Lambda$$

Thereby, the effective refractive index of the supported optical waveguide mode is represented by $n_{\mathrm{eff}}$, while the grating period is denoted by Λ, whereas m describes the integer order of the Bragg grating reflection [31]. The spectral position of $\lambda_{B}$ is consequently influenced by external parameters such as temperature or the surrounding refractive index (SRI) acting on the waveguide grating structure, either changing $n_{\mathrm{eff}}$ or Λ. Hence, a respectively induced Bragg wavelength shift represents the Bragg grating’s sensor signal. The structure’s maximum reflectivity $R_{max}$ at $\lambda_{B}$ is predominantly dictated by the grating’s RI contrast $\Delta n_{BG}$ and the grating length $l_{BG}$ after:

(2) $$R_{max}=\tanh^{2}\left(\frac{\pi }{\lambda_{B}}\Delta n_{BG}\;M_{p}\;l_{BG}\right)$$

whereas the fractional modal overlap $M_{p}$ with the grating dimensions is given by the waveguide design. Moreover, the full-width-at-half-maximum (FWHM) of the reflection signal is defined by:

(3) $$\Delta \lambda_{FWHM}=\lambda_{B}S\sqrt{{\left(\frac{\Delta n_{BG}}{2\bar{n}}\right)}^{2}+{\left(\frac{1}{N}\right)}^{2}}$$

with the mean grating RI $\bar{n}$ and the number of grating planes N. A further reflectivity factor ranges from S≈0.5 for weak gratings to S≈1 for strong gratings [32]. Therefore, the resulting Bragg reflection peak is highly dependent on the chosen grating fabrication technology in combination with the employed waveguide material’s modifiability. An optimization of $\Delta n_{BG}$ and $l_{BG}$ within the fabrication limits of the applied phase mask technique in this work is thus decisive for obtaining optimum peak tracking capability of strong, narrowband Bragg sensor signals. Carried-out investigations on Bragg grating formation in EpoCore are supported by comprehensive optical grating and waveguide simulations. Finally, a reliable and highly stable sensor performance of optimized compact EpoCore-based devices is demonstrated for rapid biomedical refractive index characterizations of whole blood samples along with the detection of human serum albumin solutions by antigen-antibody-reactions on the sensor surface.

## 2. Deep UV Photosensitivity of EpoCore Thin Films

**Figure 1:**
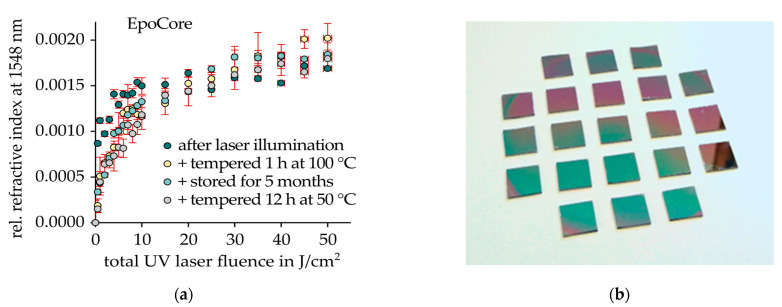
Influence of total excimer laser fluence on refractive index change of crosslinked EpoCore thin films on silicon samples. (**a**) Thermal and temporal aging steps reveal a high refractive index modification stability. (**b**) Photography of the (12 × 12) mm^2^ EpoCore on silicon samples.

The ability to locally alter the refractive index of an optical material to an adequate amount by specific optical irradiation represents a most vital property for both the fabrication of FBG and PBG concepts. This so-called photosensitivity describes RI changes due to photochemical reactions and subsequent material densification or decomposition triggered either by UV or two-photon absorption mechanisms [33]. In order to quantify the degree of 248 nm deep UV-induced refractive index modifications of cross-linked EpoCore epoxy material by the use of m-line spectroscopy, unstructured thin films with a thickness of 4 µm were prepared by spin coating EpoCore 5 photoresist solution at 3000 rpm for 60 s on standard 3” silicon wafers. After a 5-min soft bake at 90 °C, a thorough irradiation step with 200 mJ/cm² of 365 nm mercury arc lamp emission using a standard mask alignment system (EV Group EVG620) in flood exposure mode initiated photochemical reactions inside the flat negative photoresist layers. Consequently, a 5-min-long hot plate treatment at 85 °C crosslinked the exposed epoxy thin films. Afterwards, the silicon wafers were diced into 12 mm × 12 mm samples for subsequent 248 nm KrF excimer laser illumination (Coherent BraggStar S-Industrial). Thereby, the laser’s output beam was homogenized and adjusted to sample size employing a micro lens array resulting in a single-pulse laser fluence of 1 mJ/cm². The EpoCore samples were then exposed to a total laser fluence ranging from 0.5 J/cm² to 50 J/cm² corresponding to 500 and 50.000 laser pulses respectively. The induced refractive index change, i.e., the photosensitivity of EpoCore was characterized by a prism coupler m-line spectrometer (Metricon 2010/M) at the working wavelength of 1548.3 nm directly after laser illumination and after tempering all samples for 1 h at 100 °C. Furthermore, the samples were remeasured after storing the samples in a dark and dry environment for 5 months and after another temper step for 12 h at 50 °C as depicted in Figure 1. A positive and non-linear index modification with a maximum amount of Δn = (1.8 ± 0.2) × 10^−3^ was observed, which remained highly stable even after thermal aging and long-term storage procedures. All four measured sample conditions exhibited a similarly strong RI increase at low-total laser fluences up to 10 J/cm².

Above this threshold value, further RI increase moderated to a notably smaller amount. No thermal or temporal degradation of Δn was observed. Therefore, these experimental results revealed a significant stability of the induced RI modifications representing an imperative property for robust and reliable epoxy Bragg grating sensor elements in practical use. Moreover, the initially steep RI modification curve of the epoxy polymer at lower UV doses offers a wide scope for the design of both weakly and strongly reflecting Bragg grating elements.

## 3. Fabrication of Epoxy Waveguide Bragg Gratings

### 3.1. Photolithography Process

**Figure 2:**
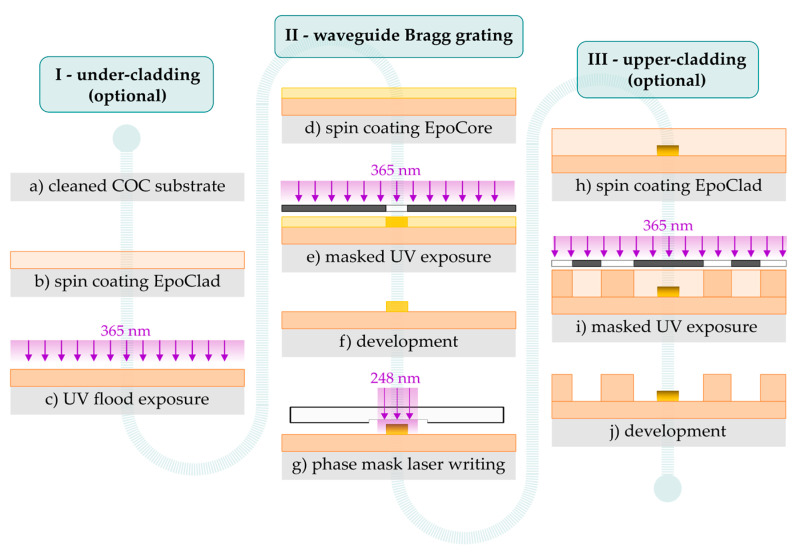
Required photolithography process steps for the fabrication of epoxy-based waveguide Bragg grating sensors.

Figure 2 provides an overview of the required main steps of the photolithography process for the fabrication of precisely structured all-polymer waveguide Bragg gratings. At first, a cleaned Topas 6017 COC substrate (a) is prepared by washing polymer sheets with a thickness of 1 mm in acetone and isopropyl alcohol (IPA). After blow drying with nitrogen, the polymer surface is activated by O_2_ plasma treatment (Diener Femto), improving wettability and adhesion of subsequently spin coated EpoClad 10 photoresist solution at 3000 rpm for an approximate thickness of 10 µm (b). The formed layer is soft baked for 5 min at 120 °C on a hotplate before 365 nm UV flood exposure with a total dose of 350 mJ/cm² (c). The EpoClad film as an optional waveguide under-cladding is finished by a post-exposure-bake for 5 min at 120 °C. The fabrication of the functional waveguide Bragg grating elements is in turn initiated by O_2_ plasma activation of the present polymer surface. The EpoCore photoresist solution is spin coated with a precisely chosen rotational speed to achieve the desired waveguide thickness (d). After soft baking for 5 min at 90 °C, the EpoCore layer is UV exposed with 200 mJ/cm² at 365 nm through an amplitude mask in contact mode defining waveguide widths of 10 µm and 15 µm (e).

Again, a post-exposure-bake for 5 min at 85 °C crosslinks the illuminated epoxy material, leaving behind the optical strip waveguide with a rectangular cross-section after selectively dissolving unirradiated EpoCore in mrDev600 (micro resist technology) developer bath for 60 s (f). Bragg gratings are locally inscribed into the waveguides via 248 nm KrF excimer laser irradiation at 20 Hz through +1/−1-phase masks (Ibsen Photonics) with grating periods around 1000 nm and maximum grating lengths of 10 mm (g).

To create a further, symmetrical upper-cladding layer on top of the waveguide structures, another EpoClad layer is processed equally to the under-cladding scheme (h). However, employed amplitude masks during the 365 nm exposure step enable a precise micro-structuring of this cladding layer as well (i). Since EpoClad still exhibits a sticky surface after soft bake, UV exposure must be performed in proximity mode. Fabrication is completed by developing the thus defined EpoClad structures for 180 s in mrDev600 (j). Finally, a manual permanent optical fiber coupling to the mechanically polished waveguide facet using optical adhesive NOA76 (Norland) enables standard interrogation (Micron Optics sm125) of the Bragg grating elements around 1550 nm. Despite inevitably introduced coupling losses in dependence of the fabricated waveguide’s dimensions, a sufficiently strong and highly stable Bragg reflection monitoring is still provided by accurate central and close coupling of the waveguide facets. The formed optical interconnection remains reliable in the long term under normal room and experimental handling conditions.

### 3.2. Phase Mask Simulation and Bragg Grating Inscription

**Figure 3:**
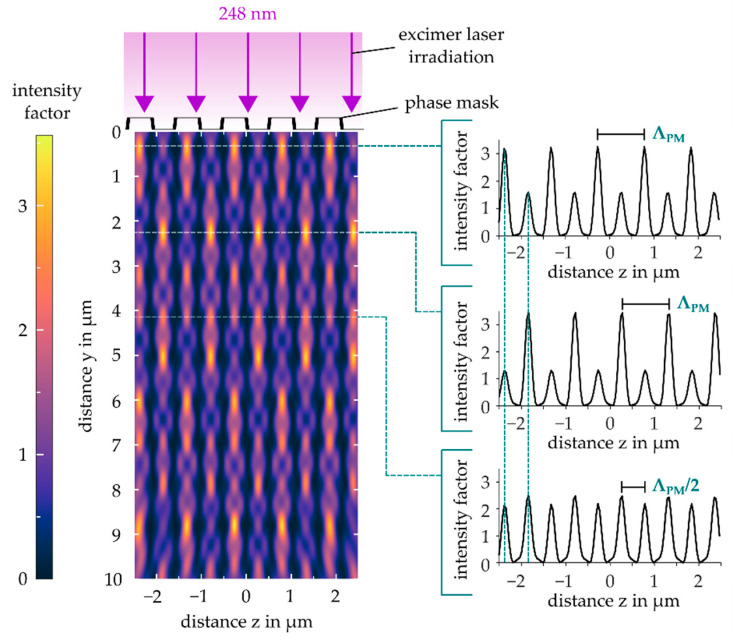
Simulated phase mask near-field (using Lighttrans VirtualLab) for a grating period of 1053 nm and an optimized grating depth of 265 nm.

The employed transmission +1/−1-phase mask design for direct Bragg grating inscription is intended to use the +1 and −1 diffraction orders only to produce an ideal sinusoidal near-field interference pattern for the specific laser wavelength of 248 nm. However, real phase masks are flawed by non-vanishing amounts of zero order and higher order diffraction. Diffraction efficiency is thereby mainly dictated by the etching depth of the grooves in the fused silica block surface forming the optical grating. An optical diffraction parameter run simulation (LightTrans VirtualLab) for a grating period of 1053 nm (50% duty cycle) was performed to identify the optimum grating depth of 265 nm yielding maximum +1/−1 order efficiency of ~37% and minimum zero and higher order efficiencies <1%. Furthermore, the corresponding near-field interference pattern was simulated in Figure 3 revealing distance-dependent occurrence of the intensity maxima. Three characteristic intensity cross-sections illustrate possibly appearing grating formations in the photosensitive epoxy material, producing either a dominant Bragg grating period equal to the phase mask’s period $\Lambda_{\mathrm{pm}}$ or $\Lambda_{\mathrm{pm}}/2$. Thereby, the phase mask’s precise orientation as well as the waveguide thickness and the 248 nm penetration depth in EpoCore represent critical Bragg grating fabrication parameters. However, according to equation 1 with respectively applied diffraction order m, both types of induced grating periods result in an identical Bragg wavelength at the desired working wavelength. Despite its non-ideal characteristics, the phase mask technique is thus a robust and reliable tool for direct laser inscription of Bragg grating elements.

**Figure 4:**
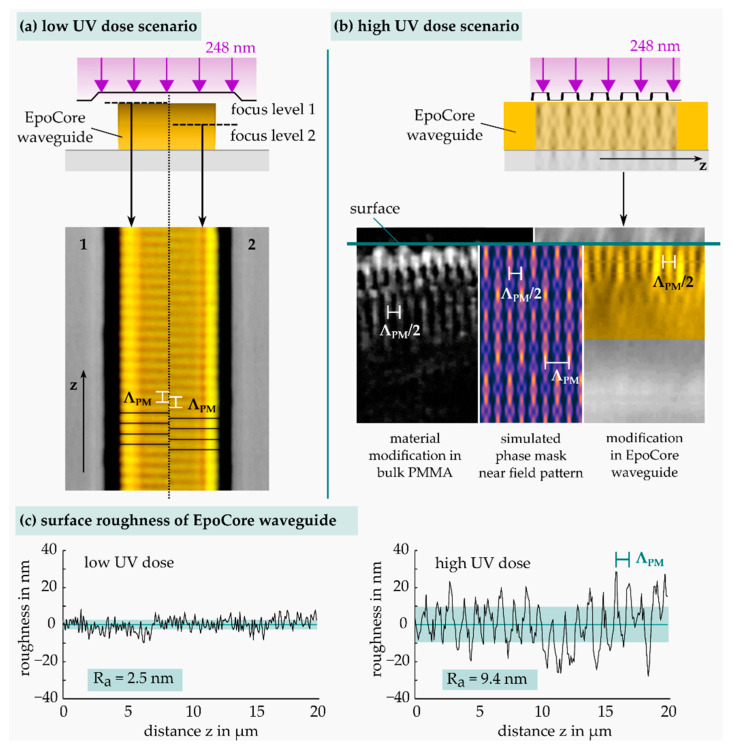
Bragg grating formation: comparison of low (10 J/cm²) and high (40 J/cm²) UV dose effects on surface and bulk volume of EpoCore waveguides irradiated through a phase mask.

In Figure 4, the distinctive influence of the totally impinged deep UV fluence on the phase mask assisted formation of EpoCore waveguide Bragg gratings is displayed. The irradiated EpoCore waveguides (marked in yellow) with a thickness of 4 µm were investigated by differential interference contrast (DIC) light microscopy and laser scanning microscopy (LSM). In the lower UV dose regime ≤10 J/cm², grating types with a characteristic grating period of $\Lambda_{\mathrm{pm}}$ were inscribed and exhibited depth-dependent π-shifts of the grating structure (see waveguide top view micrograph in Figure 4a) in excellent accordance with the described near-field simulation pattern of a real phase mask in Figure 3. Consequently, only strong primary intensity maxima of the phase mask near-field pattern contribute to an observable RI modification of the EpoCore material, while secondary maxima evidently fall below a modification threshold.

However, applying significantly higher deep UV doses >10 J/cm² result in modification contribution of the weaker secondary intensity maxima as well. In this case, Bragg gratings with a grating period of $\Lambda_{\mathrm{pm}}/2$ are fabricated in the epoxy bulk location directly below the surface (see cross sectional waveguide micrograph in Figure 4b). A comparative grating formation behavior was confirmed in photosensitive PMMA bulk polymer, resembling the intensity maxima of the phase mask near-field pattern in a similar way. A strongly decreasing modification strength with increasing material depth was observable, which corresponds to the material-dependent penetration depth of the 248 nm laser pulses. Moreover, an additional grating structure with a grating period of $\Lambda_{\mathrm{pm}}$ was implied at the surface, indicating occurring material ablation by the highly dosed laser irradiation.

Employing surface topography analysis by LSM, both irradiation cases of epoxy waveguides were investigated regarding surface roughness. A total UV laser fluence of 10 J/cm² did not cause a measurable surface corrugation in comparison with unirradiated EpoCore surfaces. In consequence, the fabricated Bragg gratings act as pure refractive index gratings inside the epoxy waveguide. In contrast, a much higher deep UV dose of 40 J/cm² induced additional periodical surface indentations with a grating period of $\Lambda_{\mathrm{pm}}$ due to laser ablation at the high-intensity maxima in the near-field pattern. The Bragg reflections of these kind of waveguide structures therefore result from a combination of the formed surface and bulk grating parts with different periods.

## 4. Online Monitoring of Bragg Signal Formation

**Figure 5:**
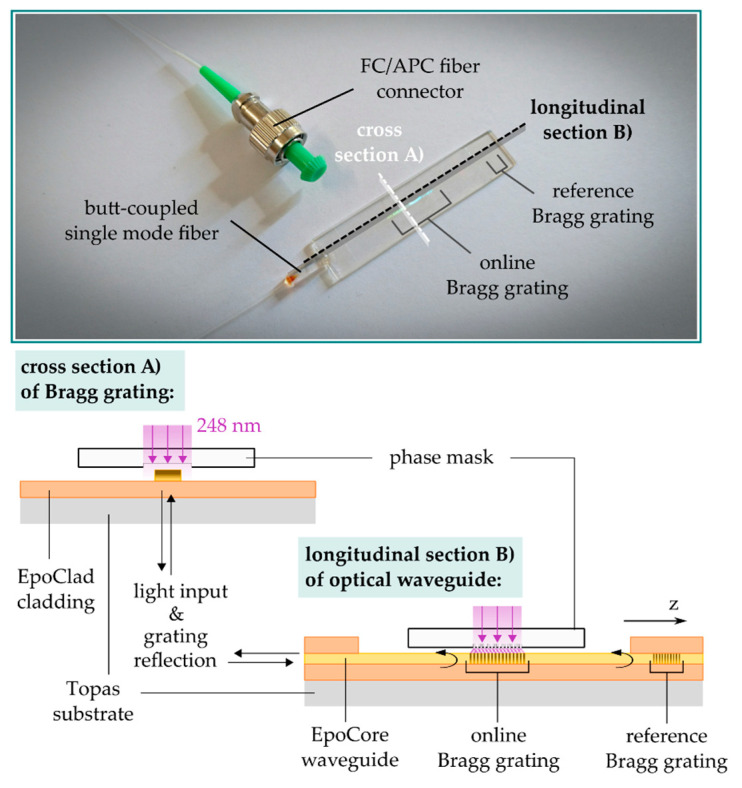
Schematic for monitoring the reflection signal behavior of stepwise deep UV induced Bragg gratings in EpoCore waveguides.

To identify optimum excimer laser parameters for the inscription of Bragg gratings in EpoCore, 50 mm long waveguides (15 µm width, 5 µm height) were prepared on an EpoClad layer as depicted in Figure 5. At the waveguide end, a short 4 mm long reference grating (Λ = 990 nm) encapsuled in EpoClad ensured a permanently attached fiber-to-chip coupling position, providing a high fundamental mode Bragg signal quality. An online-monitored Bragg grating was inscribed with increasing total laser irradiation into the waveguide center using a +1/−1-phase mask with $\Lambda_{\mathrm{pm}}$ = 1008 nm and a grating length of 10 mm. The successively rising reflection peak was characterized in terms of peak strength, FWHM, peak position as well as Bragg grating attenuation, i.e., the grating insertion loss, which is determined by decreasing peak strength of the reference grating.

**Figure 6:**
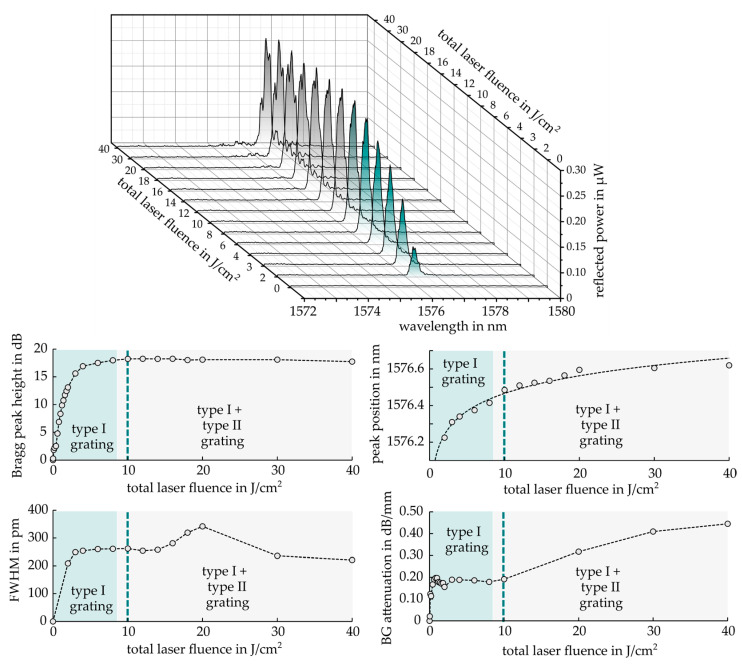
Bragg grating signal formation due to ongoing excimer laser irradiation.

The results of the parameter tracking procedure are summarized in the diagrams of Figure 6. At a low total laser fluence of 2 J/cm², a symmetric Bragg reflection peak around 1576.2 nm already appeared. With ongoing irradiation, an initially steep increase of the Bragg peak height occurred until 5 J/cm², which eventually saturated around 10 J/cm². FWHM showed a similar behavior, indicating an increasing grating contrast Δn of the forming Bragg structure with a constant length of 10 mm. BG attenuation soared up quickly, even at a very weak UV impact to a stable 0.19 dB/mm up to an applied fluence of 10 J/cm². Meanwhile, the peak position and thus the effective refractive index rose continuously, suggesting an offset-like average refractive index increase of the whole irradiated EpoCore structure, i.e., an elevated mean grating RI $\bar{n}$. This tendency together with the unchanging peak strength, however, implies a persistent grating contrast. Considering the delayed but growing influence of the less intense secondary maxima from the near-field pattern (Figure 3) by means of the found EpoCore RI modification curve (Figure 1), a constantly lasting grating contrast is explicable. By exceeding a total laser fluence of 10 J/cm², a significantly increasing BG attenuation was observable due to the beginning ablation mechanisms. Additional surface gratings were formed, leading to extra scatter loss. Furthermore, the Bragg reflection progressively tended to split into several peaks evoking a rising FWHM as well.

Therefore, the total laser fluence employed for grating formation can be divided into two parts. In the lower dose regime of up to 10 J/cm², type I gratings (marked in green), i.e., refractive index gratings in the EpoCore material were fabricated. Above 10 J/cm², type I as well as type II gratings, i.e., surface damage gratings induced by ablation were formed (marked in grey). Taking all evaluated parameters into account, a total UV laser fluence of 10 J/cm² is proposed for inscribing low-loss Bragg gratings with strong and narrow-band reflection signals.

## 5. Comparison of Measured and Simulated Bragg Reflections

**Figure 7:**
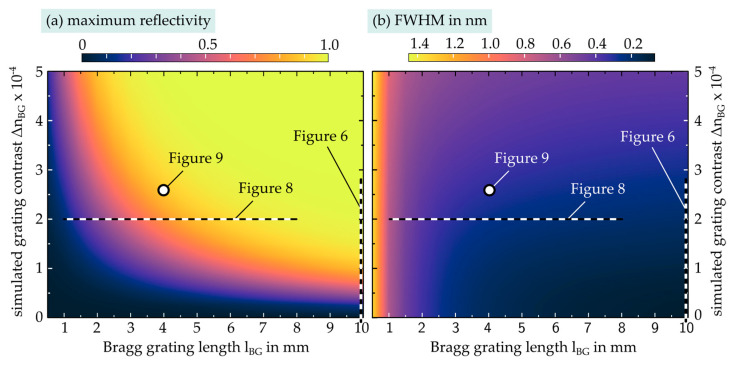
Simulated maximum reflectivity and full-width-at-half-maximum values (using RSoft GratingMOD) and the corresponding positions of carried out measurements.

To estimate the attainable phase mask assisted grating contrast in EpoCore, which mainly determines maximum grating reflectivity and FWHM, comprehensive optical grating simulations in strip waveguides were carried out using RSoft GratingMOD software. In Figure 7, the resulting influence of the grating length $l_{BG}$ (1 to 10 mm) as well as the influence of the grating contrast (0 to 5 × 10^−4^) on maximum reflectivity and FWHM is summarized in terms of heat maps. Apparently, strong Bragg reflections principally occur with a grating contrast above 1 × 10^−4^ at long grating length. However, a higher grating contrast allowed for shorter gratings of several mm only to reach comparable Bragg reflection strength.

In turn, the FWHM significantly increases in the case of a short, strong grating design. Hence, for precisely detectable, sufficiently narrow EpoCore Bragg grating signals for sensor purposes, minimum grating lengths $l_{BG}$ ≥ 2 mm are advised.

**Figure 8:**
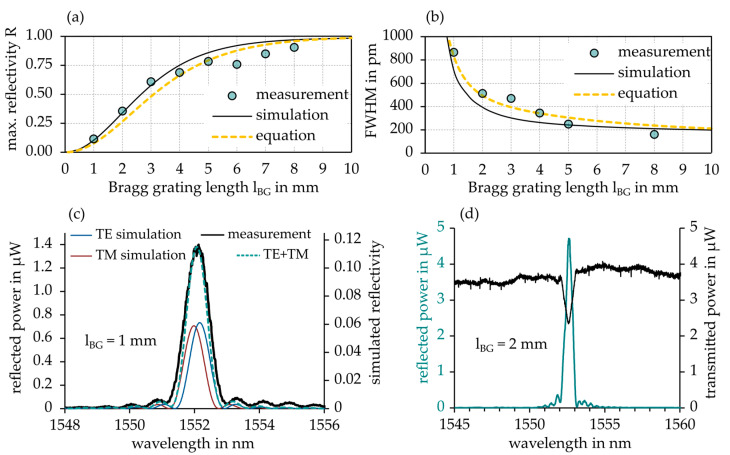
Influence of grating length on (**a**) maximum reflectivity and (**b**) the full-width-at-half-maximum (FWHM). Measurements are in good agreement with simulation data and theoretical equation. (**c**) compares simulated and measured peak appearance for a grating length of 1 mm. (**d**) exemplifies the determination of maximum grating reflection for a grating length of 2 mm.

By fitting the measured FWHM data from Figure 6 ($l_{BG}$= 10 mm) with the simulated heatmap data in Figure 7, a maximum grating contrast of $\Delta n_{\mathrm{BG}}$ = 2.8 × 10^−4^ was determined. The results of a real grating length experiment (1 mm to 8 mm in steps of 1 mm) with $\Lambda_{\mathrm{pm}}$ = 990 nm and a total laser fluence of 8 J/cm² are shown in Figure 8. By measuring the strength of the respective spectral transmission power dips $T_{d}$ (in dB) at the Bragg wavelength, maximum grating reflectivity was calculated by:

(4) $$R_{max}=1-10^{\frac{-T_{d}}{10}}$$

Figure 8a,b show grating length dependent $R_{max}$ und FWHM measurement results, which conform well to the optical simulation data and theoretical parameter equations 2 and 3 (yellow curves). In Figure 8c, the measured Bragg peak for a grating length of 1 mm is compared to simulated data for fundamental transverse electric (TE) and transverse magnetic (TM) mode reflection of the according Bragg structure which compose the overall resulting Bragg reflection. Again, an extraordinary agreement to the optical simulation result was found. Figure 8d exemplifies the measurement of the transmission dip at the Bragg wavelength for a grating length of 2 mm. The measured parameters correspond to an achieved grating contrast of $\Delta n_{\mathrm{BG}}$ = 2.0 × 10^−4^ in Figure 7.

**Figure 9:**
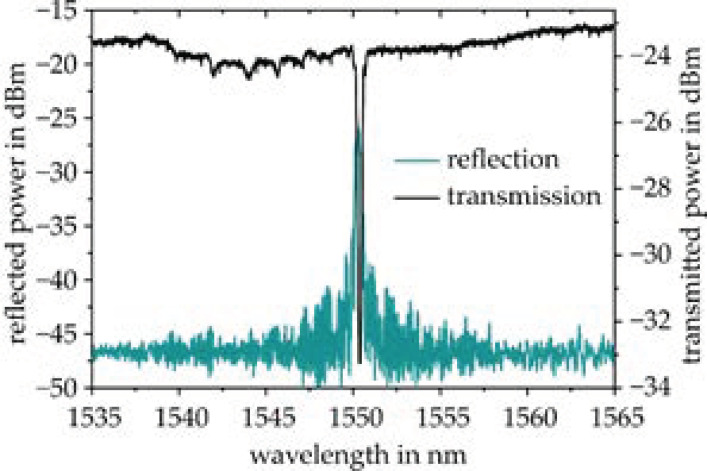
Optimized reflectivity of ~87% for a 4 mm short epoxy waveguide Bragg grating sensor with low insertion loss.

In Figure 9, a representative appearance of a strong ($R_{max}$ = 87%) and narrow-band (FWHM = 310 nm) Bragg reflection is depicted for optimized fabrication parameters of F = 10 J/cm² and $l_{BG}$ = 4 mm, which is utilized for EpoCore-based Bragg grating sensor applications. The characteristic Bragg reflection relates to an overall grating contrast of $\Delta n_{\mathrm{BG}}$ = 2.6 × 10^−4^ as shown in Figure 7.

## 6. Biomedical Application Potentials of Epoxy-Based Bragg Grating Sensors

Due to the high stability of crosslinked EpoCore material and its validated hemocompatibility [29], the optical epoxy polymer structures generally offer great potential in biomedical sensing. To employ the polymer waveguide Bragg gratings as efficient refractive index sensors, particular surrounding RI sensitivity-enhancing modifications of the waveguide layout are applied while maintaining the found optimum grating fabrication parameters. First, the waveguide height on Topas 6017 substrate is reduced to ≤1 µm near the fundamental mode cut-off (as a consequence of the asymmetric waveguide design). Furthermore, a thin high-index TiO_2_ coating of about 75 nm is sputtered onto the waveguide Bragg grating surface, as described in detail in [30], to increase evanescent wave portions in the sample media.

**Figure 10:**
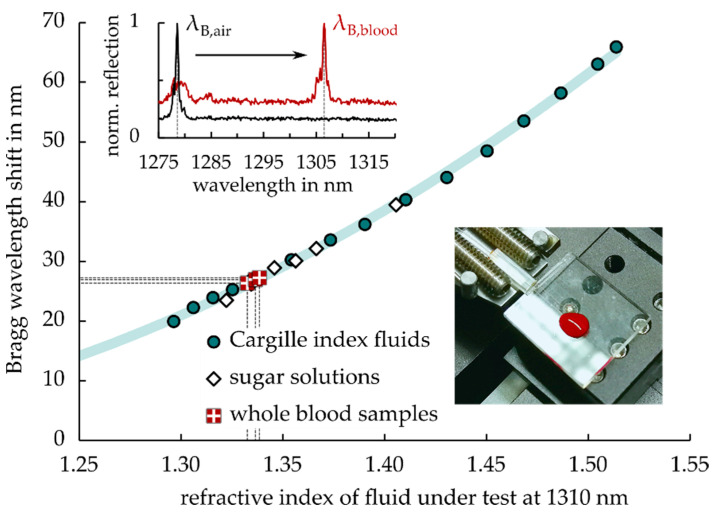
Refractive index measurement of whole blood samples using sensitized epoxy-based Bragg grating sensors. The inset exemplifies monitored Bragg wavelength shifts due to blood droplet exposure.

In a first application, a thus-modified epoxy Bragg sensor chip is demonstrated as a bulk refractive index sensor for the characterization of whole blood samples in Figure 10.

Additionally, for this particular use, a successful transfer of the former fabrication findings at 1550 nm to a working wavelength around 1310 nm is shown. A broadband light source (Thorlabs S5FC1021S SLED) in combination with a circulator and spectrometer (Ibsen I-MON 512) served as interrogation system for sensor chips with grating periods of 840 nm. A highly reproducible sensor calibration curve with remarkable Bragg wavelength shifts of up to 65.9 nm was recorded using Cargille index fluids in the RI range 1.2964–1.5137 at 1310 nm on top of the sensor grating area. Likewise, a characterization of sugar solutions verified reliable sensor performance by revealing a beneficial resistance against moisture influence. SRI sensitivity amounted to 195 nm/RIU for n = 1.4 and 295 nm/RIU for n = 1.5. By this method, three droplets of whole blood were classified to individual refractive indices of 1.3366, 1.3386, and 1.3325, respectively. The accurately determined RI of whole blood or blood plasma potentially serves as a general health indicator.

Commonly, for a selective detection and quantification of individual blood and blood plasma components, highly specific binding layers are mandatory. To qualify the employed epoxy Bragg grating sensors for basic biomedical screening purposes, selective binding of essential human serum albumin (HSA) protein to the optical sensor’s surface via antigen-antibody-reaction was evaluated. For this purpose, the Bragg grating areas were uniformly exposed to a highly concentrated (360 µg/mL) anti-HSA antibody (DuoSet ELISA DY1455, R&D Systems, Minneapolis, MN, USA) solution in phosphate buffered saline (PBS) for 45 min. By intense washing with a buffer solution (0.05% Tween^®^ 20 in PBS, Sigma Aldrich Chemie GmbH, Taufkirchen, Germany), all residual unbound antibodies were removed efficiently. After gently drying the sensor samples by nitrogen purge, a blocking step was introduced by exposing the sensor surfaces to bovine serum albumin (BSA) solution (0.1% BSA in PBS) for 30 min. Thereby, BSA serves to passivate the unoccupied and hence unspecific binding-sites of the waveguide grating surfaces and does not corrupt the functional antibody coating. After repeated washing and drying, the epoxy-based Bragg grating sensors were prepared for defined exposure to the targeted HSA solutions.

**Figure 11:**
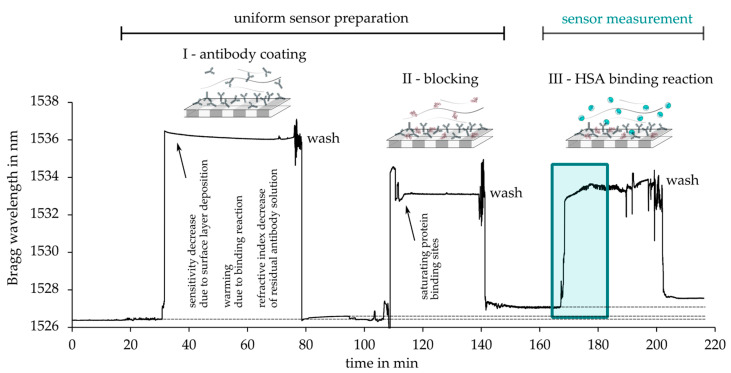
Exemplary full temporal sensor response during antibody-antigen-assay for human serum albumin detection (antigen sensing task is highlighted in green box).

Figure 11 demonstrates the full temporal, characteristic Bragg wavelength behavior during sensor preparation and selective HSA binding. All these sensor signals were monitored by standard interrogation system in the wavelength range around 1550 nm.

**Figure 12:**
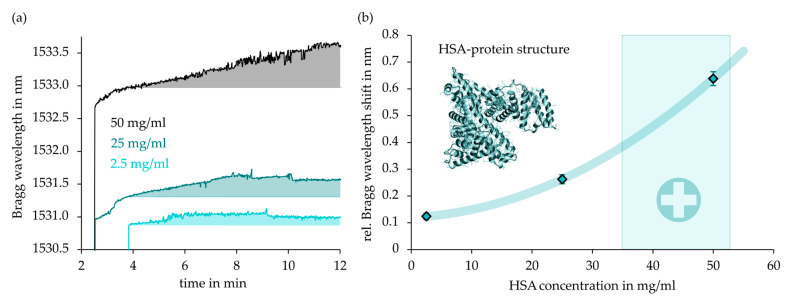
Comparative sensor response in dependence on HSA concentration. (**a**) temporal sensor response. (**b**) determined Bragg wavelength shifts.

Large and instantaneous Bragg wavelength shifts are induced by the bulk refractive index of the applied fluids themselves, while surface binding reactions in general cause a small, gradual alteration of the sensor signal’s spectral position until saturation. Thus, for the detection of selective HSA binding and to investigate the influence of HSA concentration, the gradients of the induced Bragg wavelength shifts evoked by different HSA concentrations of 2,5 mg/mL, 25 mg/mL, and 50 mg/mL were evaluated accordingly. Figure 12a shows the HSA concentration-dependent sensor responses in comparison. A distinct and continuous positive Bragg wavelength shift was observed during the exposure of the three different measurand concentrations. After twelve minutes of exposure, all Bragg sensor signals were in a stable condition indicating saturation state. In Figure 12b, the Bragg wavelength differences at the well-detectable reference points at 4 min and 12 min of HSA exposure were consulted as a sensitivity measure (average of two measurements). The obtained curve resembled the exponential shape sensitivity against the surrounding refractive index of the raw, non-selective epoxy-based Bragg grating sensor. Especially, the experimental results attested to the optical sensor design considerable sensitivity near healthy-state HSA concentrations (33–52 mg/mL) in human blood plasma [34], suggesting highly valuable biomedical rapid test applications.

Since all the employed sensor chips originated from the same fabrication batch (with the same waveguide and coating thickness), very high measurement reproducibility using different sensor chips was obtained throughout the experiments. Moreover, with respective thorough cleaning and drying procedures of the individual sensor surface after each measurement, a high measurement repeatability was given as well.

## 7. Conclusions

In this work, we extensively investigated the nature of deep UV-induced Bragg grating formation in EpoCore strip waveguides by the use of the popular phase mask technique. The laser fluence dependent refractive index modifications of the epoxy material in combination with the complex near field diffraction patterns of non-ideal phase masks strongly affected the resulting grating type. Optimum grating fabrication parameters for compact, long-term stable, strong, and narrowband Bragg reflections in EpoCore were derived with exceptional agreement to optical grating simulations. Moreover, a modified waveguide grating design based on all preliminary findings was employed for highly sensitive surrounding refractive index determination with sensitivities up to 295 nm/RIU for sample media with n = 1.5. Attractive biomedical application potential for rapid characterization of whole blood samples as well as for the specific determination of human serum albumin were demonstrated successfully. Consequently, an optimized Bragg sensor implementation into future optofluidic lab-on-a-chip devices in combination with innovative, low-cost wavelength interrogation schemes (as for example in [35]) is strongly encouraged.

## Data Availability

The data presented in this study are available on request from the corresponding author.
